# Suicide-Related Knowledge among Italian Early Career Psychiatrists and Trainees: Results from a Cross-Sectional Survey

**DOI:** 10.3390/brainsci12121619

**Published:** 2022-11-25

**Authors:** Isabella Berardelli, Andrea Aguglia, Emanuele Cassioli, Francesco Saverio Bersani, Luisa Longo, Mario Luciano, Amedeo Minichino, Jacopo Santambrogio, Marco Solmi, Rodolfo Rossi, Michele Ribolsi, Eleonora Gattoni, Alessio Maria Monteleone

**Affiliations:** 1 Department of Neurosciences, Mental Health and Sensory Organs, Faculty of Medicine and Psychology, Suicide Prevention Centre, Sant’Andrea Hospital, Sapienza University of Rome, Via di Grottarossa, 1035, 00189 Rome, Italy; 2 Department of Neuroscience, Rehabilitation, Ophthalmology, Genetics, Maternal and Child Health, Section of Psychiatry, University of Genoa, 16132 Genoa, Italy; 3 Istituto di Ricovero e Cura a Carattere Scientifico Ospedale Policlinico San Martino, 16132 Genoa, Italy; 4 Psychiatry Unit, Department of Health Sciences, University of Florence, 50134 Florence, Italy; 5 Department of Human Neurosciences, Sapienza University of Rome, 00185 Rome, Italy; 6 Psychiatry and Clinical Psychopharmacology University Unit, Department of Mental Health, Azienda Sanitaria Locale (ASL) Latina, 04100 Latina, Italy; 7 Department of Psychiatry, Johns Hopkins University School of Medicine, Baltimore, MD 21201, USA; 8 Department of Psychiatry, University of Campania “L. Vanvitelli”, 80138 Naples, Italy; 9 Department of Psychiatry, University of Oxford, Oxford OX1 2JD, UK; 10 School of Medicine and Surgery, University of Milano-Bicocca, 20900 Monza, Italy; 11 Department of Psychiatry, University of Ottawa, Ottawa, ON K1N 6N5, Canada; 12 Department of Mental Health, The Ottawa Hospital, Ottawa, ON K1Z 7K4, Canada; 13 Ottawa Hospital Research Institute (OHRI), Clinical Epidemiology Program, University of Ottawa, Ottawa, ON K1Z 7K4, Canada; 14 School of Epidemiology and Public Health, Faculty of Medicine, University of Ottawa, Ottawa, ON K1N 6N5, Canada; 15 Department of Child and Adolescent Psychiatry, Charité Universitätsmedizin, 10117 Berlin, Germany; 16 Department of Systems Medicine, University of Rome Tor Vergata, 00133 Rome, Italy; 17 Unit of Neurology, Neurophysiology, Neurobiology and Psychiatry, Department of Medicine, University Campus Bio-Medico of Rome, 00128 Rome, Italy; 18 Fondazione Policlinico Universitario Campus Bio-Medico, 00128 Roma, Italy; 19 Psychiatry Ward, Maggiore della Carità University Hospital, 28100 Novara, Italy

**Keywords:** suicide prevention, suicide knowledge, suicide skills, training professionals

## Abstract

The training of mental health professionals is an important component of suicide-prevention programs. A cross-sectional survey was conducted in different Italian regions to evaluate knowledge of, and attitudes toward, suicide as well as the experience of a patient’s suicide or a suicide attempt in early career psychiatrists (ECPs) and trainees (N = 338). The Suicide Knowledge and Skills Questionnaire and the Impact of a Patient’s Suicide on Professional and Personal Lives scale were administered. Furthermore, symptoms of intrusion, avoidance, and arousal were examined through the Impact of Event Scale in ECPs and trainees who had experienced the suicide of a patient or a suicide attempt. Participants with training were more confident in the clinical management of suicide-risk patients. The group with experience of a patient’s suicide reported more suicide skills except for support and supervision. Finally, the participants who reported a patient’s suicide presented a more conservative patient selection, difficulties in relationships, loss of self-esteem, dreams linked to suicide, intrusive thoughts of suicide, guilt, and anger. Our results show that knowledge of, and attitudes toward, suicide are essential in the management of suicide-risk patients.

## 1. Introduction

Suicide is a notable public health issue [1]; in Italy, approximately 4000 people die by suicide every year and suicide rates vary in different Italian geographic areas [2,3]. The assessment of patients with suicidal risk (suicidal ideation and attempts) represents an essential skill for early career psychiatrists (ECPs) and trainees, due to the significant increase of suicidal behaviors in general and psychiatric population [4,5]. Moreover, training for mental health professionals is essential in the field of suicide prevention [6,7,8,9,10,11]. Only half of the psychiatrists’ trainees and ECPs had received adequate and focused training on suicide risk and prevention during their professional education [12,13,14]. Several authors reported a relationship between the ability of psychiatrists to recognize patients at risk of suicide and inadequate knowledge of the potential signs and risk factors of suicide [15,16]. In this framework, improved knowledge and attitudes about suicide risk may allow ECPs and trainees to better identify patients with suicidal ideation and suicidal attempts, increasing suicide-prevention strategies. To our knowledge, only a few papers investigated knowledge and attitudes toward suicide among mental health professionals [17,18]. Jiao et al. compared knowledge and attitudes toward suicide in a sample of 187 psychiatrists from different psychiatric hospitals in Shanghai versus 548 urban community members [17]. Results showed that psychiatrists presented more stigmatizing beliefs about suicide, although they considered suicide an important social concern. Furthermore, Erbuto and coworkers demonstrated that healthcare professionals, who reported a patient’s suicide, had more suicide skills and an increased tendency to hospitalize suicide-risk patients in a psychiatric unit than professionals who had not reported a patient’s suicide [18].

Approximately half of all psychiatrists have reported a patient’s suicide during their career [19,20]. The suicide of a patient could produce important emotional consequences for psychiatrists, including anxiety, guilt, frustration, impotence, depression, and high levels of stress [20]. Moreover, psychiatrists could present negative attitudes toward patients with suicidal ideations and suicidal attempts [21]. Barman et al. demonstrated the presence of trauma and stress symptoms after a patient’s suicide in a sample of 292 psychiatrists and 31 trainees [22]. On the other hand, Castelli Dransart et al. pointed out that a patient’s suicide could offer an opportunity for professional growth, increasing the awareness of suicide knowledge and requiring more supervision [23].

Based on the hypothesis that suicide-prevention strategies are influenced by psychiatrists’ suicide-related knowledge and attitudes [24,25], the primary aim of this survey was to assess knowledge and attitudes toward suicide in a representative sample of Italian ECPs and trainees in psychiatry. We conducted a cross-sectional investigation to evaluate the relationship between suicide knowledge and skills, suicide training and the experience of a patient’s suicide or a suicide attempt in ECPs and trainees in psychiatry in different Italian regions. In addition, the presence of symptoms of intrusion, avoidance, and arousal in ECPs and trainees in psychiatry who experienced a suicide of a patient or a suicide attempt was also examined.

## 2. Materials and Methods

### 2.1. Sample

A cross-sectional survey on trainees in psychiatry and ECPs in different Italian regions was developed. The online survey was carried out during the month of December 2021, using the same methodology provided for other previous studies published by the ECP network of the Italian Society of Psychopathology (SOPSI-GG) [26,27]. The online survey was disclosed between January and March 2022. A self-administered, anonymous questionnaire in survey monkey was developed after several online meetings with the other SOPSI-GG members. IB and AA carried out the first draft of the survey. The other members of SOPSI-GG (eleven ECPs from different Italian regions), over two weeks, revised the online questionnaire and checked the clarity of the questionnaire, monitoring the time required to complete the survey and confirming the inclusion and exclusion criteria of the study. The survey was then revised in line with the comments reported in the meetings. The SOPSI-GG members held two different online meetings to discuss and review the survey. The mean duration was approximately 15 min to complete the survey. All the members of SOPSI-GG invited Italian ECPs to participate in the present study through email or other technological support. Since the members of SOPSI-GG belong to different Italian regions, the survey was mainly disseminated in the regions to which the members themselves belong. The National and International Psychiatric Associations provided the following definition of ECPs: psychiatrists under 40 years of age or with fewer than 5 years of clinical practice after specialization [28]. The inclusion criteria were to be a trainee in psychiatry or an ECP in Italy (based on self-declaration), as the principal target of this investigation. Exclusion criteria were other medical specialties and professional figures in the field of mental health or not being under 40 years old or with fewer than 5 years of clinical practice after specialization. The SOPSI-GG members declared that the questionnaire was anonymous, and that all personal data were protected. Furthermore, the SOPSI-GG members did not know the participants’ identities. Informed consent was obtained from participants when they started the survey. All the procedures are in line with the ethical standards of the relevant national and institutional committees on human experimentation and with the Helsinki Declaration of 1975, revised in 2013 [29]. This study did not involve animals or vulnerable subjects, e.g., patients. The research did not impose risks, harm, or disadvantage to the participants. and there was not a prospective evaluation of the participants. This study was reviewed and approved by the University Department (Department of Neurosciences, Mental Health and Sensory Organs) local ethics committee.

### 2.2. Assessment

An ad-hoc schedule to collect sociodemographic characteristics and involvement in research activities was administered, including the assessment for professionals with experience of a patient’s suicide.

The Suicide Knowledge and Skills Questionnaire (SKSQ) [30] is a 13-item questionnaire, and it is used to investigate knowledge and perceived competence in the management of patients at suicidal risk. This questionnaire consists of two subscales: suicide knowledge and suicide skills. Responses are based on a 5-point Likert scale, from completely agree to completely disagree. Two previous studies [30,31] reported low (Cronbach’s alpha inferior to 0.70) and acceptable (Cronbach’s alpha between 0.81 and 0.84) internal consistency for the suicide-knowledge and the suicide-skills subscales, respectively. The miscellaneous nature of the items of the suicide-knowledge subscale could explain the low internal consistency, which is also present in this study (Cronbach’s alpha was 0.48). Cronbach’s alpha for the suicide-skills-confidence subscale was 0.80 while the Cronbach’s alpha of SKSQ without the differentiation on subscales was 0.64.

The Impact of a Patient’s Suicide on Professional and Personal Lives scale is a questionnaire used only for psychiatrists with an experience of a patient’s suicide [32]. Responses were based on a Likert scale, ranging from 1 (minimum impact) to 7 (maximum impact). No statistical validity or reliability of the scale was performed, because the authors used the items as part of a survey. In line with previous studies, only responses to single items were analyzed [32,33]. Therefore, the 5-point Likert scale responses to the suicide-skills subscale were converted as follows: the responses “strongly disagree- disagree” and the responses “agree” and “strongly agree” were associated, while the 7-point Likert scale responses of the Impact of a Patient’s Suicide on Professional and Personal Lives scale were merged as follows: 1 to 3 as “disagree”, 5 to 7 as “agree”, and 4 as “neither”. In the present sample, Cronbach’s alpha was 0.91.

The Impact of Event Scale (IES) was used to evaluate the presence of post-traumatic stress symptomatology during the last seven days [34]. The IES consists of 15 items with a 4-point scale (0 = not at all, 1 = rarely, 3 = sometimes and 5 = often). A total sum score for the IES varies from 0 to 75. A score of ≥35 is considered as presenting post-traumatic stress symptomatology. In the present sample, Cronbach’s alpha was 0.90.

### 2.3. Statistical Analysis

All analyses were performed with the Statistical Package for Social Sciences (SPSS 27.0). Sociodemographic and clinical characteristics are presented either as mean ± standard deviation (SD) or as absolute counts and percentages for continuous and categorical variables, respectively. They were assessed for normal distribution, using the Kolmogorov–Smirnov test. First, our sample was divided into two subgroups, based on the presence of training on suicide-risk received, investigating differences in suicide-skills- and knowledge-subscale items. The second step of the analysis was based on statistical differences in suicide-skills- and knowledge-subscale items, according to the experience of the patient’s suicide: in this case, three subgroups were identified (none versus suicide attempt versus suicide). Finally, the different impact between suicide attempt and suicide was investigated. Therefore, a series of ANOVAs, Fisher’s exact tests, and chi-square (χ^2^) tests were used for bivariate analyses. In the case of non-normal distribution, the Mann–Whitney test was used. Bonferroni post hoc tests were used for group comparison. Finally, a multinomial regression analysis was performed to assess the predictive power of sociodemographic characteristics on the suicide-related variables.

Statistical significance was set for *p*-values < 0.05.

## 3. Results

### 3.1. Sample Characteristics

A total of 338 ECPs and trainees in psychiatry completed the survey. The questionnaire was not returned by all the ECPs invited to participate in the study; 90 ECPs and trainees in psychiatry did not complete the survey and were not included in the study. Of the whole sample, 40 were ECPs and 298 were trainees in psychiatry. Most of the respondents were women (N = 190; 56.2%), with a mean age of 30.1 (SD = 3.6) years. Regarding the working areas, we divided the sample into three different working areas: northern Italy (Lombardy, Piedmont, Emilia-Romagna, Friuli Venezia Giulia, Trentino-South Tyrol, and Veneto), central Italy (Lazio, Marche, Tuscany, Abruzzo, and Umbria), and southern Italy (Basilicata, Calabria, Campania, Molise, Sardinia, and Sicily). Overall, 129 psychiatrists were from northern Italy (38.2%), 127 from central Italy (37.6%), and 82 from southern Italy (24.2%). Of the whole sample, 207 respondents (61.2%) received training on suicide risk, while 131 (38.8%) did not (Table 1).

### 3.2. Differences between Subgroups with and without Training on Suicide Risk

The two subgroups differed according to age (t = −4.20, *p* < 0.001), experience of a patient’s suicide attempt or suicide (Fisher’s exact test *p* = 0.001), and some perceived skills and knowledge of suicide. The groups did not differ for sex and working area.

Specifically, the respondents that received training on suicide risk were older (30.7 ± 3.7 versus 29.0 ± 3.2, *p* < 0.001) and reported more experiences of a patient’s suicide attempt or suicide than the group that did not receive training (72.9% versus 55.0%, *p* = 0.001).

Regarding the questions about the perceived skills on suicide, the participants with training were more likely to agree with the statement: “I have received the training I need to engage and assist those with suicidal desire” (2.9 ± 1.0 versus 2.3 ± 0.9; *p* < 0.001), “I have the skills I need to engage those with suicidal desire and/or intent” (2.8 ± 1.0 versus 2.3 ± 0.9; *p* < 0.001), and “I am comfortable asking direct and open questions about suicide” (3.5 ± 1.0 versus 3.1 ± 1.0; *p* = 0.001), but notably no difference was found for the statement “I have the SUPPORT/SUPERVISION I need to engage and assist those with suicidal desire” (see Table 2).

**Figure 1:**
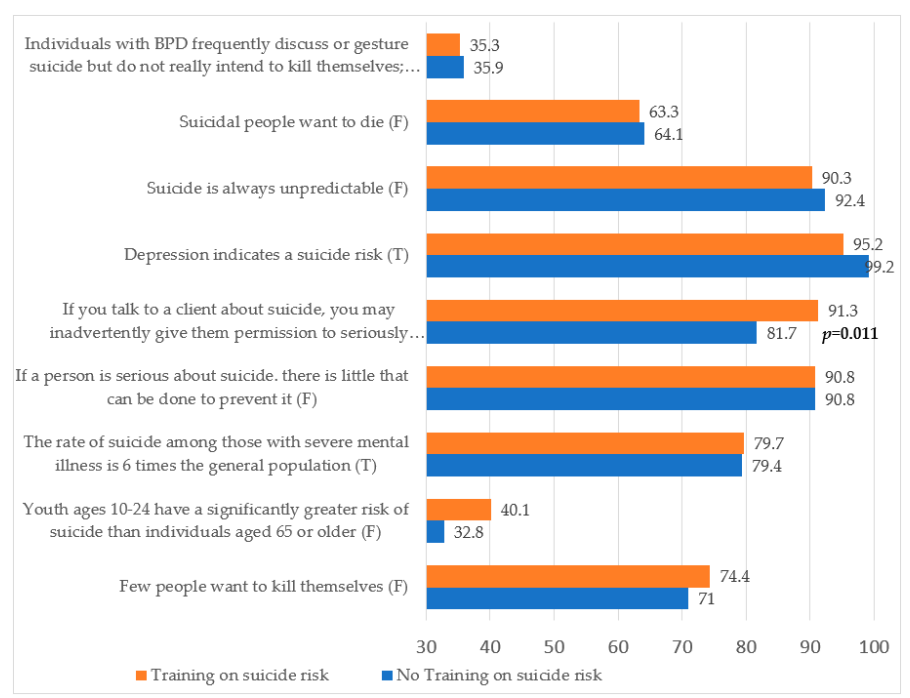
Differences between subgroups according to training on suicide risk on suicide-knowledge-subscale item. (BPD = Borderline Personality Disorders).

Finally, a difference was found for the knowledge section regarding the statement “If you talk to a client about suicide, you may inadvertently give them permission to seriously consider it”, in which participants with training were more likely to answer correctly than participants that had not received training on suicide risk (91.3% versus 81.7%, *p* = 0.011) (See Figure 1).

### 3.3. Differences between Groups according to the Experiences of Patient’s Suicide

The sample was then split into three subgroups, according to the experience of a patient’s suicide (none, patient with a suicide attempt, and patient with a completed suicide). The subgroups differed for age (F = 9.48), working area (χ^2^ = 16.86), and several questions about suicide skills. The groups did not differ according to sex and questions about suicide knowledge.

**Figure 2:**
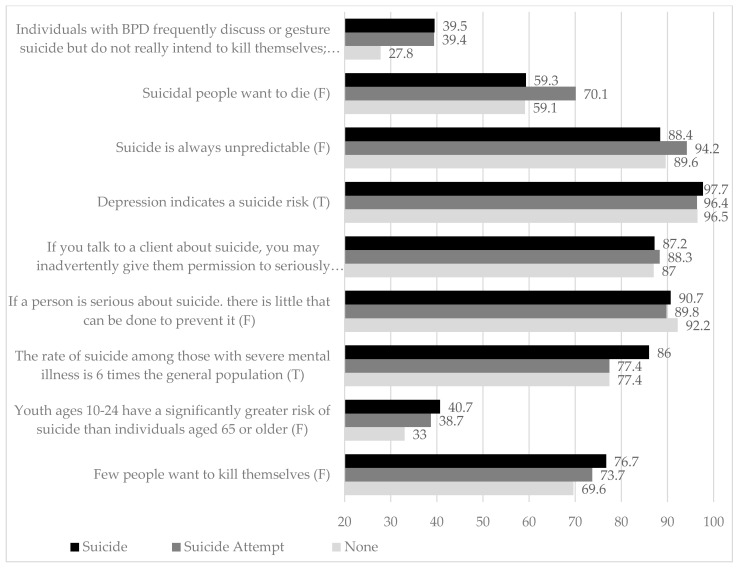
Differences between subgroups according to the experience of patient’s suicide.

Participants that experienced a patient’s suicide were older (suicide experience 31.5 ± 3.6, suicide attempt 29.6 ± 3.1, and none 29.5 ± 3.7), and more likely to work in northern Italy (52.3% versus 35.8% and 30.4%, for central and southern Italy, respectively). Moreover, the group with experience of patient’s suicide reported more suicide skills, except for the statement “I have the SUPPORT/SUPERVISION I need to engage and assist those with suicidal desire” (See Table 3 and Figure 2).

### 3.4. Differences between the Impact of Patient’s Suicide Attempt vs. Patient’s Suicide

Subsequently, we focused on the impact of a patient’s suicide attempt or suicide (see Table 4). The participants that experienced a patient’s suicide reported more conservative patient selection (2.5 ± 1.6 versus 2.0 ± 1.3, *p* = 0.034), disturbed relationships with friends (1.9 ± 1.2 versus 1.5 ± 0.9, *p* = 0.018), loss of self-esteem (2.7 ± 1.6 versus 1.9 ± 1.3, *p* < 0.001), dreams related to suicide (2.0 ± 1.5 versus 1.6 ± 1.0, *p* = 0.020), intrusive thoughts of suicide (2.1 ± 1.7 versus 1.6 ± 1.1, *p* = 0.023), guilt (3.1 ± 1.7 versus 2.2 ± 1.4, *p* < 0.001), and anger (2.9 ± 1.9 versus 2.2 ± 1.5, *p* = 0.001). Moreover, those who experienced a patient’s suicide reported more avoidance symptoms at the subscale of the IES (M = 6.80) than participants who experienced patient’s suicide attempt (6.8 ± 6.6 versus 5.0 ± 5.7, *p* = 0.031) (See Table 5).

## 4. Discussion

Results of the present study suggest that ECPs and trainees in psychiatry, that carried out training on suicide, were more confident in the clinical management of suicide-risk patients, confirming the importance of suicide knowledge in recognizing and managing patients at risk of suicide [7,10]. Furthermore, results showed that the subgroup of clinicians with experience of a patient’s suicide presented more suicide skills except for support and supervision. Finally, participants who experienced a patient’s suicide reported several important clinical and psychological consequences.

Our findings showed that 61.2% of participants received training on suicide risk. Furthermore, clinicians receiving training on suicide risk were older and reported more experiences of a patient’s suicide attempt or completed suicide than the subgroup without any training. Regarding the questions about perceived skills on suicide, participants with training were more confident in their knowledge and attitudes toward suicide risk and in the clinical management of patients at higher risk of suicide. In particular, they were more confident in asking direct and open questions about suicide. Furthermore, only one difference was found for the knowledge section, regarding the statement “If you talk to a client about suicide, you may inadvertently give them permission to seriously consider it”, in which clinicians with training were more likely to answer correctly than participants without training on suicide risk. These results underlined the link between knowledge in the field of suicidology and myths about suicide. According to Joiner, there are many myths about suicide, including the fact that if you talk about suicide, you can stimulate a suicide attempt in a patient. In *Myths About Suicide*, the author underlined that, with better knowledge about suicide, we can increase all suicide-prevention strategies [35]. Our data show the importance of specific suicide theoretical training, suggesting that knowing how to manage patients with suicide risk was one of the most important learning needs of trainees in psychiatry. Although training through clinical cases and congresses was the preferred learning modality [26], these data show that theoretical education is also important.

When dividing the whole sample into three different subgroups, according to the experience of a patient’s suicide (none, suicide attempt, and patient suicide, respectively), results showed that participants that experienced a patient’s suicide were older and more likely to work in northern Italy. These results are in line with evidence that, in Italy, the northern region presents the highest suicide rates and, therefore, psychiatrists working in this area are more likely to experience a patient’s suicide [36,37]. Furthermore, the subgroup with experience of a patient’s suicide reported more suicide skills, except for the statement “I have the SUPPORT/SUPERVISION I need to engage and assist those with suicidal desire” (See Table 3), showing the need for more support and supervision after the death of a patient.

Finally, we focused on the impact of a patient’s suicide attempt or suicide on healthcare professional well-being (See Table 4). The participants who reported a patient’s suicide presented a more conservative patient selection, disturbed relationships, loss of self-esteem, dreams about suicide, intrusive thoughts, and feeling of guilt and anger. Moreover, those who experienced a patient’s suicide reported more avoidance symptoms on the subscale of the IES than participants who experienced a patient’s suicide attempt. The loss of a patient by suicide produces an important emotional impact on psychiatrists, particularly on ECPs and trainees in psychiatry [32]; in fact, trainees in psychiatry reported high rates of a patient’s suicide [38]. Gibbons et al., in a survey on 174 psychiatrists, found that, after a patient’s suicide, clinicians showed emotional and clinical distress [39]. Recently, McCutcheon et al., in a survey on 43 residents in psychiatry, underlined the necessity of improving clinicians’ coping strategies following a patient’s suicide [40].

This study has several strengths and limitations. This is the first survey aimed at exploring the knowledge and attitudes about suicide in a representative sample of Italian ECPs and trainees in psychiatry. Further strengths are the high representativeness of the sample, characterized by the homogeneous geographic distribution, participants working in different clinical settings and the use of validated rating scales, even if the use of self-reporting questionnaires could be biased by under-reporting, under-estimating and misunderstanding the issues. As a first limitation, the investigation through an online survey could imply a potential recruiting bias, with the self-selection of more technological ECPs. The sample involved in the study is not homogeneous (of the whole sample, 40 were ECPs and 298 were trainees in psychiatry). Furthermore, although the whole sample could be considered representative, the questionnaire was not returned by all the ECPs: it was distributed to at least 450 participants. Additionally, several answers, especially those concerning attitudes toward suicide, could be affected by a potential affective-state bias, rather than reflecting the real attitudes and knowledge in daily clinical practice, although the survey was conducted anonymously. Moreover, the IES scale was administered only to those ECPs and trainees who experienced a suicide attempt or suicide ideation of a patient, and we did not collect the information if the event took place before or after the training. Furthermore, the cross-sectional design of the present survey does not allow the investigation of the temporal correlation among the assessed variables. Finally, to estimate potential confounders or effect modifiers of responses, several clinical variables, including personality traits, previous personal environmental experiences, psychological or temperamental characteristics, as well as coping strategies and resilience, were not considered.

## 5. Conclusions

This study investigated an issue of public-health interest, providing important information on training needs of trainees in psychiatry and ECPs, in the field of suicide prevention. A patient’s suicide may have emotional and professional impact on young psychiatrists. Overall, our results show that increased knowledge and improved attitudes about suicide are important factors for suicide-prevention strategies. Assessing and treating suicidal patients should be an educational priority for ECPs and trainees in psychiatry.

Finally, we suggest the importance of carrying out targeted training on suicide prevention that gives young psychiatrists the theoretical knowledge to assess the various and different suicide-risk factors and how they interact with each other, as well as the pharmacological skills to improve the use of anti-suicidal drugs in suicide-risk patients.

## Figures and Tables

**Table 1:** Sample characteristics.

	Whole Sample (N = 338)
Gender (Females)	190 (56.2%)
Age (M ± SD)	30.1 ± 3.6
Working area	
Northern Italy	129 (38.2%)
Central Italy	127 (37.6%)
Southern Italy	82 (24.2%)
Lifetime training on suicide risk	207 (61.2%)
12-month lifetime training on suicide risk	136 (40.2%)

**Table 2:** Differences between subgroups according to training on suicide risk (general characteristics and suicide-skills subscale items).

Mean ± SD/N (%)	No Training on Suicide Risk (N = 131)	TRAINING on Suicide Risk (N = 207)	t/χ^2^	*p*
Sex (Females)	78 (59.5%)	112 (54.1%)		0.368 ^a^
Current Age (years)	29.0 ± 3.2	30.7 ± 3.7	−4.20	<0.001
Working area			2.92	0.232
Northern Italy	49 (37.4%)	80 (38.6%)		
Central Italy	44 (33.6%)	83 (40.1%)		
Southern Italy	38 (29.0%)	44 (21.3%)		
Experience of patient’s suicide attempt or suicide	72 (55.0%)	151 (72.9%)		0.001 ^a^
Suicide Skills Subscale Items				
I have received the TRAINING I need to engage and assist those with suicidal desire	2.3 ± 1.0	2.9 ± 1.0	−5.22	<0.001
I have the SKILLS I need to engage those with suicidal desire and/or intent	2.3 ± 0.9	2.8 ± 1.0	−5.58	<0.001
I have the SUPPORT/SUPERVISION I need to engage and assist those with suicidal desire	3.1 ± 1.1	3.2 ± 1.1	−0.71	0.477
I am comfortable asking direct and open questions about suicide	3.1 ± 1.0	3.5 ± 1.0	−3.34	0.001

^a^ = Non-parametric test for statistical comparison was performed.

**Table 3:** Differences between subgroups according to the experience of patient’s suicide (general characteristics and suicide-skills-subscale items).

Mean ± SD/N (%)	None (N = 115)	Suicide Attempt (N = 137)	Suicide (N = 86)	χ^2^/F	*p*
Sex (Females)	66 (57.4%)	76 (55.5%)	48 (55.8%)	0.101	0.951
Current Age (years)	29.5 ± 3.7	29.6 ± 3.1	31.5 ± 3.6	9.48	<0.001 C > A, B
Working area				16.86	0.002
Northern Italy	35 (30.4%)	49 (35.8%)	45 (52.3%)		
Central Italy	55 (47.8%)	45 (32.8%)	27 (31.4%)		
Southern Italy	25 (21.7%)	43 (31.4%)	14 (16.3%)		
Suicide Skills Subscale Items					
I have received the TRAINING I need to engage and assist those with suicidal desire	2.4 ± 1.0	2.6 ± 1.1	3.0 ± 1.1	7.17	<0.001 C > A
I have the SKILLS I need to engage those with suicidal desire and/or intent	2.4 ± 0.9	2.6 ± 1.0	3.0 ± 1.0	9.85	<0.001 C > A, B
I have the SUPPORT/SUPERVISION I need to engage and assist those with suicidal desire	3.2 ± 1.0	3.2 ± 1.1	3.1 ± 1.1	0.52	0.597
I am comfortable asking direct and open questions about suicide	3.2 ± 1.0	3.3 ± 1.0	3.6 ± 0.9	4.69	0.010 C > A

**Table 4:** Multinomial regression evaluating the predictive power of sociodemographic characteristics on the suicide-related variables.

Variables	B	E.S.	Wald	*p*	Exp (B)	95% CI for EXP
Patients with Suicide Attempts						
Current age	−0.006	0.041	0.022	0.882	0.994	0.918–1.077
Northern Italy	−0.309	0.361	0.731	0.393	734	0.362–1.491
Central Italy	−0.825	0.348	5.612	0.018	0.438	0.221–867
Southern Italy	0.0 *	.	.	.	.	.
Patients committing suicide						
Current age	0.154	0.042	13.470	<0.001	1.167	1.075–1.267
Northern Italy	1.042	0.452	5.317	0.021	2.835	1.169–6.876
Central Italy	0.039	0.456	0.007	0.931	1.040	0.426–2.543
Southern Italy	0.0 *	.	.	.	.	.

Category of reference: No suicide; * = this parameter is set to zero because it is redundant.

**Table 5:** Differences between experience of patient’s suicide attempt versus suicide.

Mean ± SD	Suicide Attempt	Suicide	U	*p*
1. Increased attention to legal aspect of practice	4.4 ± 1.9	4.6 ± 1.7	5549	0.459
2. Increased tendency to hospitalize	3.4 ± 1.6	3.6 ± 1.5	5346	0.237
3. More conservative patient selection	2.0 ± 1.3	2.5 ± 1.7	4951	0.034
4. Increased focus on suicide cues	5.0 ± 1.7	5.2 ± 1.6	5359.5	0.248
5. Increased concerns with death issues	4.2 ± 1.8	4.7 ± 1.6	4986.5	0.050
6. Increased use of collegial consultation	3.9 ± 1.9	4.3 ± 1.9	5184.5	0.127
7. More conservative record keeping	3.6 ± 1.9	3.8 ± 1.8	5471.5	0.365
8. Increased use of peer consultation	4.6 ± 1.8	4.6 ± 1.6	5881.5	0.984
9. Disturbed relationships with colleagues	1.7 ± 1.1	2.1 ± 1.6	5251	0.130
10. Disturbed relationships with friends	1.5 ± 0.9	1.9 ± 1.2	4941.5	0.018
11. Loss of self-esteem	1.9 ± 1.3	2.7 ± 1.6	4056.5	<0.001
12. Dreams related to suicide	1.6 ± 1.0	2.0 ± 1.5	4962	0.020
13. Disturbed relationships with family	1.5 ± 0.9	1.9 ± 1.5	5199	0.076
14. Intrusive thoughts of suicide	1.6 ± 1.1	2.1 ± 1.7	4986.5	0.023
15. Guilt	2.2 ± 1.4	3.1 ± 1.7	4039	<0.001
16. Anger	2.2 ± 1.5	2.9 ± 1.9	4452.5	0.001
17. Emotional numbness	1.7 ± 1.2	2.0 ± 1.5	5508.5	0.346
18. Social withdrawal	1.5 ± 1.0	1.8 ± 1.4	5279	0.099
Impact of Event Scale				
– Intrusion	4.3 ± 5.0	5.8 ± 6.4	5076	0.078
– Avoidance	5.0 ± 5.7	6.8 ± 6.6	4892	0.031

## Data Availability

The data presented in this study are available on request from the corresponding author. The data are not publicly available, due to privacy/ethical restrictions.
